# Association between dietary patterns and lipid profile in patients with non-alcoholic fatty liver disease: a retrospective cross-sectional study

**DOI:** 10.3389/fnut.2026.1794910

**Published:** 2026-05-21

**Authors:** Bijun Chen, Huifa Zhou, Ying Tang

**Affiliations:** 1 The People’s Hospital of Baiyun District, Guangzhou, China; 2 Zhujiang Hospital, Southern Medical University, Guangzhou, China

**Keywords:** balanced diet, ketogenic diet, lipid profile, low-fat diet, non-alcoholic fatty liver disease (NAFLD)

## Abstract

**Background:**

Dietary composition significantly influences metabolic health, particularly lipid metabolism and liver function.

**Objective:**

This study aimed to examine the associations between ketogenic, low-fat, and balanced dietary patterns with lipid parameters, liver enzymes, and renal function in the Chinese adult population.

**Methods:**

This retrospective cross-sectional study analyzed data from 650 adults categorized into 3 dietary groups based on recorded dietary intake: ketogenic (*n* = 210), low-fat (*n* = 220), and balanced (*n* = 220). Demographic, socioeconomic, anthropometric, clinical, and biochemical variables were assessed. Group comparisons were performed using a one-way analysis of variance (ANOVA) and chi-square tests, while a multivariable linear regression analysis was used to evaluate associations between dietary patterns and metabolic outcomes.

**Results:**

Baseline demographic and socioeconomic characteristics were comparable across the groups. The ketogenic diet was associated with higher levels of total cholesterol, triglycerides, low-density lipoprotein cholesterol (LDL-C), and high-density lipoprotein cholesterol (HDL-C) compared with the low-fat and balanced diets (*p* < 0.01). Higher alanine aminotransferase (ALT) and aspartate aminotransferase (AST) levels were also observed in association with the ketogenic diet. In adjusted analyses, the ketogenic diet remained significantly associated with increases in total cholesterol (*β* = +62.9 mg/dL), triglycerides (*β* = +36.5 mg/dL), LDL-C (*β* = +34.2 mg/dL), HDL-C (*β* = +5.6 mg/dL), ALT (*β* = +8.7 U/L), and AST (*β* = +5.9 U/L) (all *p* ≤ 0.001). No significant associations were observed between dietary patterns and renal function markers, including serum creatinine and estimated glomerular filtration rate (eGFR).

**Conclusion:**

The ketogenic diet was associated with higher HDL-C and increased atherogenic lipids and liver enzymes; however, no significant association was observed with renal function. These findings highlight the importance of individualized dietary guidance and metabolic monitoring.

## 1 Introduction

Non-alcoholic fatty liver disease (NAFLD) is a global public health issue that is growing in prevalence (1). It is characterized by excess fat accumulation in hepatocytes in the absence of significant alcohol consumption (2). Due to obesity, insulin resistance, dyslipidemia, and metabolic syndrome, NAFLD is associated with an increased risk of type 2 diabetes and cardiovascular disease (3). Patients with NAFLD commonly exhibit altered lipid profiles, including elevated total cholesterol, LDL-C, and triglycerides, with variable changes in HDL-C (4, 5). Dietary modification is commonly used to treat NAFLD because it affects liver fat deposition, insulin sensitivity, and total lipid metabolism (6, 7). The ketogenic diet has gained attention for its potential effects on weight reduction and glucose regulation (8). The ketogenic diet is rich in fat, moderate in protein, and low in carbohydrates. However, its effects on lipid metabolism in NAFLD patients remain inconsistent across studies (9). Some studies have reported improvements in triglycerides and HDL-C, whereas others have shown increases in total cholesterol and LDL-C (10). These latter findings raise concerns about potential long-term cardiovascular safety. Low-fat diets, which are beneficial for dyslipidemia and heart health (11, 12), reduce fat intake and increase carbohydrate intake (13). These diets may be associated with lower LDL-C and total cholesterol, but their effects on HDL-C and triglycerides depend on the types of carbohydrates and fats consumed (14). Balanced diets, containing moderate proportions of carbohydrates, proteins, and fats along with whole grains, fruits, vegetables, and lean protein, support long-term metabolic stability and reduce cardiometabolic risk (15). However, limited comparative evidence exists on the differential effects of ketogenic, low-fat, and balanced diets on lipid profiles in NAFLD patients in real-world clinical settings. To optimize dietary outcomes and reduce the risk of liver and cardiovascular complications, it is important to understand how ketogenic, low-fat, and balanced diets influence lipid profiles in individuals with NAFLD. Therefore, this study aimed to evaluate the associations between dietary patterns and lipid parameters in NAFLD patients. In addition, liver enzymes and metabolic indicators were assessed to provide a comprehensive evaluation of metabolic outcomes.

## 2 Materials and methods

### 2.1 Study design and setting

A retrospective cross-sectional study was conducted to examine the association between ketogenic, low-fat, and balanced dietary patterns and lipid profiles in patients with NAFLD. Data were collected from January to June 2025 using computerized medical records, clinical databases, dietary assessments, and liver registries from Baiyun District People’s Hospital and its affiliated campuses. All participating centers followed standardized clinical protocols for patient evaluation and laboratory investigations to ensure consistency across sites.

### 2.2 Sample size and population detail

A total of 650 adult patients with NAFLD were enrolled. Patients were selected using convenience sampling from the hospital’s computerized database. Based on reported food intake, the study population was divided into three diet groups: ketogenic (*n* = 210), low-fat (*n* = 220), and balanced (*n* = 220). Based on recorded dietary macronutrient intake, participants were categorized into ketogenic, low-fat, and balanced diet groups.

#### 2.2.1 Inclusion criteria

Participants were eligible if they met the following criteria:

1. Possessed comprehensive dietary intake records, encompassing macronutrients, micronutrients, salt, and added sugars.
2. Had available metabolic and hepatic outcome data, including liver enzymes (ALT, AST, ALP, and GGT), lipid profiles (total cholesterol, triglycerides, LDL-C, HDL-C), and other pertinent laboratory indicators.
3. Had recorded clinical information on comorbidities and medication use available.

#### 2.2.2 Exclusion criteria

Patients were excluded if they had:

1. Congenital liver diseases not related to NAFLD.
2. Chronic systemic illnesses that could significantly impair liver function (e.g., cirrhosis from other causes or end-stage renal disease).
3. Incomplete dietary or clinical records.

### 2.3 Data collection tool and procedure

Data were extracted using a standardized structured abstraction form developed from validated dietary and NAFLD literature sources. The tool was reviewed by subject experts for content validity and pilot-tested on a subset of records to ensure clarity, consistency, and inter-rater reliability. Variables collected included the following: demographic and anthropometric characteristics: age, sex, height, weight, BMI, and waist circumference; clinical factors: comorbidities such as diabetes, hypertension, dyslipidemia, and medication intake; dietary factors: total energy intake, macronutrient distribution, saturated and trans-fat intake, dietary fiber, fruit and vegetable intake, and added sugar consumption, obtained from validated dietary assessment records; and metabolic and hepatic outcomes: lipid profile (total cholesterol, triglycerides, LDL-C, HDL-C, and non-HDL cholesterol), liver enzymes (ALT, AST, ALP, and GGT), and glycemic parameters [fasting glucose, fasting insulin, and Homeostatic Model Assessment of Insulin Resistance (HOMA-IR)].

### 2.4 Classification of dietary patterns

Dietary intake data were obtained from clinical nutrition records, including validated food frequency questionnaires (FFQs) and 24-h dietary recall data. Daily energy and macronutrient intake (carbohydrates, proteins, and fats) were calculated using standard food composition tables. Participants were then categorized into dietary pattern groups based on predefined macronutrient distribution ranges. Specifically, individuals were classified as following: a ketogenic diet if carbohydrate intake was <50 g/day (approximately <10% of total energy), fat contributed was ≥60–75% of total daily energy, and protein intake ranged between 15 and 30% of total energy. Participants were assigned to the low-fat diet group if total fat intake contributed ≤25% of total daily energy, with carbohydrate intake typically ranging from 55 to 65% and protein intake ranging from 15 to 20% of total energy. Those categorized in the balanced diet group had macronutrient distributions within the range of 45–55% carbohydrates, 20–25% protein, and 25–30% fat. In cases where dietary intake overlapped between categories, classification was based on the predominant macronutrient contributing the highest proportion of total energy intake. All dietary classifications were independently verified by a trained clinical nutritionist to ensure consistency and minimize misclassification bias (16). These groups were used to assess associations between dietary patterns and serum lipid parameters in patients with NAFLD.

### 2.5 Clinical and laboratory measurements

Anthropometric and biochemical parameters, including lipid profiles, liver enzymes, and renal function markers, were obtained from clinical records. Laboratory measurements were performed in accredited hospital laboratories using standardized assay protocols, ensuring comparability across centers, although a small degree of inter-laboratory variability could not be entirely excluded.

### 2.6 Covariates and confounding variables

Multivariable regression models were adjusted for key confounding factors, including age, sex, body mass index (BMI), diabetes status, hypertension, smoking status, and medication use (including lipid-lowering and antidiabetic drugs where available). Data on physical activity were not consistently available for all participants and, therefore, were not included in the final analysis, which may introduce residual confounding.

### 2.7 Ethical considerations

The Ethics Committee of Baiyun District People’s Hospital approved this research (Approval Number: B-2022-031-01) in accordance with the Declaration of Helsinki. This study was a retrospective cross-sectional study; therefore, informed consent was not required. To maintain anonymity, all patient records were anonymized.

### 2.8 Statistical analysis

We analyzed the data with SPSS 26. Continuous variables were expressed as mean ± standard deviation (SD), and categorical variables were expressed as frequencies and percentages (*n*, %). The normality of continuous variables was assessed using the Shapiro–Wilk test. For comparisons among more than two dietary groups, a one-way ANOVA was applied to normally distributed variables with equal variances. Categorical variables were compared using the chi-square test, where applicable. Multivariable linear regression models were used to assess the association between dietary patterns and lipid profile parameters, adjusting for potential confounders including age, sex, BMI, comorbidities, and medication use. A *p*-value of < 0.05 was considered statistically significant. All statistical results were reported with corresponding confidence intervals, where appropriate, to ensure consistency and transparency.

## 3 Results

### 3.1 Demographic and socioeconomic characteristics of study participants

The study divided 650 participants’ records into ketogenic (210), low-fat (220), and balanced diet groups. Age differences were minor, with the ketogenic group averaging 48.3 ± 9.6 years, the low-fat 47.8 ± 10.2 years, and the balanced diet 49.1 ± 9.7 years (*p* = 0.44). Groups had similar gender distributions. Men made up 63.8% (*n* = 134) of the ketogenic, 61.8% (*n* = 136) of the low-fat, and 63.6% (*n* = 140) of the balanced diet (*p* = 0.89) groups. The ketogenic, low-fat, and balanced diet groups had 72.4% (*n* = 152), 71.8% (*n* = 158), and 73.2% (*n* = 161) urban residents, respectively (*p* = 0.94), indicating an even split between urban and rural residents (*p* = 0.94). Approximately 56.7% (*n* = 119) of the ketogenic group, 59.5% (*n* = 131) of the low-fat group, and 56.8% (*n* = 125) of the balanced group had at least a college degree (*p* = 0.73), which indicates that the education levels of the 3 groups were comparable. Employment status was also very similar among the groups (ketogenic: 67.6%, low-fat: 67.3%, balanced: 68.2%; *p* = 0.97). The average monthly income of the ketogenic group was RMB¥7,800 ± 2,100, that of the low-fat group was RMB¥8,000 ± 2,250, and that of the balanced diet group was RMB¥7,900 ± 2,180 (*p* = 0.61). No statistically significant differences in income were observed across groups. Therefore, participants’ economic status did not differ significantly. The smoking rate was nearly identical across the 3 groups: 33.8% (*n* = 71) in the ketogenic group, 34.5% (*n* = 76) in the low-fat group, and 33.6% (*n* = 74) in the balanced diet group (*p* = 0.98). Approximately 39.0% (*n* = 82) of the ketogenic group, 39.1% (*n* = 86) of the low-fat group, and 38.6% (*n* = 85) of the balanced diet group reported non-excessive alcohol intake (*p* = 0.99) (Table 1).

**Table 1:** Demographic and socioeconomic characteristics of study participants.

Variable (*n* = 650)	Ketogenic diet (*n* = 210)	Low-fat diet (*n* = 220)	Balanced diet (*n* = 220)	*p*-value
Age (years)	48.3 ± 9.6	47.8 ± 10.2	49.1 ± 9.7	0.44
Male, *n* (%)	134 (63.8)	136 (61.8)	140 (63.6)	0.89
Urban residence, *n* (%)	152 (72.4)	158 (71.8)	161 (73.2)	0.94
Education ≥ College, *n* (%)	119 (56.7)	131 (59.5)	125 (56.8)	0.73
Employed, *n* (%)	142 (67.6)	148 (67.3)	150 (68.2)	0.97
Monthly income (RMB)	7,800 ± 2,100	8,000 ± 2,250	7,900 ± 2,180	0.61
Smokers, *n* (%)	71 (33.8)	76 (34.5)	74 (33.6)	0.98
Alcohol intake (non-excessive), *n* (%)	82 (39.0)	86 (39.1)	85 (38.6)	0.99

Values are presented as mean ± SD or number (%). Continuous variables were compared using one-way ANOVA, and categorical variables using the chi-square test.

### 3.2 Anthropometric and clinical characteristics

Mean weights of the different diet groups were as follows: ketogenic diet group 78.6 ± 11.2 kg, which was slightly higher than the low-fat group (76.9 ± 10.9 kg) and balanced diet group (77.4 ± 11.1 kg) groups. Yet, the difference was not statistically significant (*p* = 0.28). The ketogenic group had a BMI of 29.6 ± 3.8 kg/m^2^, while the low-fat group had a slightly lower BMI of 28.7 ± 3.9 kg/m^2^, and the balanced group had a BMI of 29.0 ± 3.7 kg/m^2^. This difference was statistically significant (*p* = 0.03). The ketogenic group had a higher waist circumference (97.1 ± 8.6 cm) than the low-fat (95.2 ± 9.0 cm) and balanced diet (95.8 ± 8.7 cm) groups (*p* = 0.04), indicating greater abdominal fat. Blood pressure measurements were close to each other across all three groups: the ketogenic group’s average systolic BP was 132 ± 14 mmHg, the low-fat group was 130 ± 15 mmHg, and the balanced group was 131 ± 14 mmHg (*p* = 0.41). The ketogenic group’s average diastolic BP was 84 ± 9 mmHg, the low-fat group was 83 ± 10 mmHg, and the balanced group was 84 ± 9 mmHg (*p* = 0.67). No statistically significant differences were observed in blood pressure across groups. The percentages of diabetic patients were similar in all three groups: 37.1% in the ketogenic group, 37.7% in the low-fat group, and 36.4% in the balanced diet group (*p* = 0.96). Similarly, the percentages of hypertensive patients were 43.3% in the ketogenic group, 42.7% in the low-fat group, and 43.2% in the balanced diet group (*p* = 0.99). No statistically significant differences were observed in these clinical conditions (Table 2).

**Table 2:** Anthropometric and clinical characteristics.

Parameter	Ketogenic diet	Low-fat diet	Balanced diet	*p*-value
Weight (kg)	78.6 ± 11.2	76.9 ± 10.9	77.4 ± 11.1	0.28
BMI (kg/m^2^)	29.6 ± 3.8	28.7 ± 3.9	29.0 ± 3.7	0.03
Waist circumference (cm)	97.1 ± 8.6	95.2 ± 9.0	95.8 ± 8.7	0.04
Systolic BP (mmHg)	132 ± 14	130 ± 15	131 ± 14	0.41
Diastolic BP (mmHg)	84 ± 9	83 ± 10	84 ± 9	0.67
Diabetes mellitus, *n* (%)	78 (37.1)	83 (37.7)	80 (36.4)	0.96
Hypertension, *n* (%)	91 (43.3)	94 (42.7)	95 (43.2)	0.99

Values are mean ± SD for continuous variables and *n* (%) for categorical variables; *p*-values from ANOVA or chi-square test; significant if *p* < 0.05.

### 3.3 Lipid profile, liver enzymes, and renal function parameters

The ketogenic diet group (*n* = 210) had a mean total cholesterol level of 268.6 ± 34.2 mg/dL compared with the low-fat diet group (198.6 ± 29.4 mg/dL) and the balanced diet group (205.7 ± 30.6 mg/dL) (*p* < 0.001). Hence, the ketogenic diet was associated with higher cholesterol levels. Similarly, triglyceride concentration was significantly elevated in the ketogenic group (218.9 ± 56.4 mg/dL) compared with the low-fat (189.8 ± 55.6 mg/dL) and balanced diet (182.4 ± 53.9 mg/dL) groups (*p* = 0.004). For LDL-cholesterol (LDL-C), the ketogenic diet group showed a significant increase in LDL-C levels (164.8 ± 29.1 mg/dL), compared with the low-fat (124.8 ± 25.9 mg/dL) and balanced diet groups (130.6 ± 26.8 mg/dL) (*p* < 0.001). Moreover, HDL-cholesterol (HDL-C) was significantly higher in the ketogenic group (49.2 ± 7.1 mg/dL) than in the low-fat (41.6 ± 6.5 mg/dL) and balanced diets (43.7 ± 6.7 mg/dL) (*p* < 0.001). This finding indicates that cardioprotective lipoproteins were slightly increased. The ketogenic diet was associated with significantly higher liver enzyme levels, with ALT reaching 52.6 ± 20.4 U/L and AST 44.8 ± 18.1 U/L, which were statistically significant (ALT: *p* < 0.001; AST: *p* = 0.002) compared with the low-fat diet (ALT: 41.4 ± 17.6 U/L; AST: 36.5 ± 15.4 U/L) and balanced diet (ALT: 43.9 ± 18.1 U/L; AST: 38.1 ± 15.9 U/L) groups. Meanwhile, there were no significant differences in ALP levels between the groups (ketogenic: 94.2 ± 22.6 U/L; low-fat: 88.2 ± 20.9 U/L; balanced: 89.7 ± 21.3 U/L; *p* = 0.21), suggesting that cholestatic liver function was minimally affected. Regarding kidney function, the ketogenic group displayed a serum creatinine level slightly higher (0.93 ± 0.19 mg/dL) than that of the low-fat (0.89 ± 0.17 mg/dL) and balanced diet (0.90 ± 0.18 mg/dL) groups. Nevertheless, these variations were not statistically significant (*p* = 0.17). Similarly, eGFR levels were not different among the three groups (ketogenic: 91.6 ± 15.2 mL/min/1.73 m^2^; low-fat: 94.1 ± 15.1 mL/min/1.73 m^2^; balanced: 93.3 ± 14.8 mL/min/1.73 m^2^; *p* = 0.39) (Table 3).

**Table 3:** Lipid profile, liver enzymes, and renal function parameters.

Parameter	Ketogenic diet (*n* = 210)	Low-fat diet (*n* = 220)	Balanced diet (*n* = 220)	*p*-value
Total cholesterol (mg/dL)	268.6 ± 34.2	198.6 ± 29.4	205.7 ± 30.6	<0.001
Triglycerides (mg/dL)	218.9 ± 56.4	189.8 ± 55.6	182.4 ± 53.9	0.004
LDL-C (mg/dL)	164.8 ± 29.1	124.8 ± 25.9	130.6 ± 26.8	<0.001
HDL-C (mg/dL)	49.2 ± 7.1	41.6 ± 6.5	43.7 ± 6.7	<0.001
ALT (U/L)	52.6 ± 20.4	41.4 ± 17.6	43.9 ± 18.1	<0.001
AST (U/L)	44.8 ± 18.1	36.5 ± 15.4	38.1 ± 15.9	0.002
ALP (U/L)	94.2 ± 22.6	88.2 ± 20.9	89.7 ± 21.3	0.21
Serum creatinine (mg/dL)	0.93 ± 0.19	0.89 ± 0.17	0.90 ± 0.18	0.17
eGFR (mL/min/1.73 m^2^)	91.6 ± 15.2	94.1 ± 15.1	93.3 ± 14.8	0.39

Values are mean ± SD; *p*-values from ANOVA; significant if *p* < 0.05.

### 3.4 Multivariable regression analysis: association between diet type and lipid and renal outcomes

After adjusting for multiple variables, the ketogenic diet was observed to increase total cholesterol substantially and statistically (*β* = +62.9 ± 4.1 mg/dL; 95% CI: 54.9 to 70.9; *p* < 0.001), indicating a significant positive association (Table 4). The low-fat diet, however, was associated with a slight, statistically insignificant decrease in total cholesterol (*β* = −5.1 ± 3.7 mg/dL; 95% CI: −12.3 to 2.1; *p* = 0.16), indicating that it did not have a major impact on this parameter. The ketogenic diet was linked to an increase in triglycerides (*β* = +36.5 ± 5.3 mg/dL; 95% CI: 26.0 to 47.0; *p* < 0.001), indicating a significant association. The low-fat diet increased triglycerides very slightly (*β* = +3.6 ± 5.1 mg/dL; 95% CI: −6.5 to 13.7; *p* = 0.49), suggesting no change. A ketogenic diet causes a significant increase in LDL-cholesterol (LDL-C), with a mean increase of +34.2 mg/dL (SE = 3.8) and a 95% CI of 26.7 to 41.7 (*p* < 0.001). This finding indicates a significant association with higher LDL-C levels. Conversely, the low-fat diet exhibited no significant association with LDL-C (*β* = −0.3 ± 3.5 mg/dL; 95% CI: −7.2 to 6.6; *p* = 0.92). A ketogenic diet was associated with a significant increase in HDL cholesterol (HDL-C) levels (*β* = +5.6 ± 0.9 mg/dL; 95% CI: 3.8 to 7.4; *p* < 0.001). However, the low-fat diet significantly reduced HDL-C levels (*β* = −2.1 mg/dL; SE = 0.8; 95% CI: −3.7 to −0.5; *p* = 0.01), indicating a statistically significant reduction in HDL-C levels. The ketogenic diet was significantly associated with elevated ALT levels (*β* = +8.7 ± 2.1 U/L; 95% CI: 4.5 to 12.9; *p* < 0.001) and increased AST levels (*β* = +5.9 U/L; SE = 1.8; 95% CI: 2.3 to 9.5; *p* = 0.001). These findings might indicate that the liver is either vulnerable or its metabolism has been altered. The low-fat diet, however, did not significantly reduce ALT (*β* = −2.5 ± 1.9 U/L; 95% CI: −6.2 to 1.2; *p* = 0.19) or AST (*β* = −1.6 ± 1.7 U/L; 95% CI: −5.0 to 1.8; *p* = 0.36). Regarding kidney function, neither diet was strongly associated with serum creatinine levels. Although small differences in creatinine and eGFR were observed, these were not statistically significant and should be interpreted with caution (*β* = +0.02 mg/dL; SE = 0.04; 95% CI: −0.06 to 0.10; *p* = 0.61), whereas the low-fat diet showed a similar non-significant decrease (*β* = −0.01 ± 0.03 mg/dL; 95% CI: −0.07 to 0.05; *p* = 0.78). Neither diet influenced eGFR, although the ketogenic diet decreased it by 0.8 mL/min/1.73 m^2^ (SE = 1.5; 95% CI: −3.7 to 2.1; *p* = 0.64).

**Table 4:** Multivariable regression analysis: association between diet type and lipid and renal outcomes.

Outcome	Diet type	*β* ± SE	95% CI	*p*-value
Total cholesterol (mg/dL)	Ketogenic	+62.9 ± 4.1	54.9 to 70.9	<0.001
	Low-Fat	−5.1 ± 3.7	−12.3 to 2.1	0.16
Triglycerides (mg/dL)	Ketogenic	+36.5 ± 5.3	26.0 to 47.0	<0.001
	Low-Fat	+3.6 ± 5.1	−6.5 to 13.7	0.49
LDL-C (mg/dL)	Ketogenic	+34.2 ± 3.8	26.7 to 41.7	<0.001
	Low-Fat	−0.3 ± 3.5	−7.2 to 6.6	0.92
HDL-C (mg/dL)	Ketogenic	+5.6 ± 0.9	3.8 to 7.4	<0.001
	Low-Fat	−2.1 ± 0.8	−3.7 to −0.5	0.01
ALT (U/L)	Ketogenic	+8.7 ± 2.1	4.5 to 12.9	<0.001
	Low-Fat	−2.5 ± 1.9	−6.2 to 1.2	0.19
AST (U/L)	Ketogenic	+5.9 ± 1.8	2.3 to 9.5	0.001
	Low-Fat	−1.6 ± 1.7	−5.0 to 1.8	0.36
Serum Creatinine (mg/dL)	Ketogenic	+0.02 ± 0.04	−0.06 to 0.10	0.61
	Low-Fat	−0.01 ± 0.03	−0.07 to 0.05	0.78
eGFR (mL/min/1.73 m^2^)	Ketogenic	−0.8 ± 1.5	−3.7 to 2.1	0.64
	Low-Fat	+0.5 ± 1.4	−2.2 to 3.2	0.73

Reference group: balanced diet. Models were adjusted for age, sex, BMI, diabetes, hypertension, smoking status, and baseline value of the outcome variable. Confidence intervals (95% CI) are reported as lower to upper bounds.

## 4 Discussion

The ketogenic diet was significantly associated with elevated ALT levels (+8.7 U/L; SE = 2.1; 95% CI: 4.5 to 12.9; *p* < 0.001) and increased AST levels (+5.9 U/L; SE = 1.8; 95% CI: 2.3 to 9.5; *p* = 0.001). These findings indicate differential associations between dietary patterns and hepatic enzyme activity, which may reflect diet-related modulation of hepatic lipid metabolism and hepatocellular stress. The low-fat diet was not associated with statistically significant changes in ALT (*β* = −2.5 U/L; 95% CI: −6.2 to 1.2; *p* = 0.19) or AST (*β* = −1.6 U/L; 95% CI: −5.0 to 1.8; *p* = 0.36). Regarding renal outcomes, no significant associations were observed between dietary patterns and serum creatinine levels. The ketogenic diet showed a small, non-significant increase in serum creatinine (*β* = +0.02 mg/dL; SE = 0.04; 95% CI: −0.06 to 0.10; *p* = 0.61), while the low-fat diet showed a non-significant decrease (*β* = −0.01 mg/dL; SE = 0.03; 95% CI: −0.07 to 0.05; *p* = 0.78). Estimated glomerular filtration rate (eGFR) did not differ significantly between dietary groups, with the ketogenic diet showing a non-significant reduction (*β* = −0.8 mL/min/1.73 m^2^; SE = 1.5; 95% CI: −3.7 to 2.1; *p* = 0.64) and the low-fat diet showing a non-significant increase (*β* = +0.5 mL/min/1.73 m^2^; SE = 1.4; 95% CI: −2.2 to 3.2; *p* = 0.73).

Baseline demographic characteristics were comparable across dietary groups, minimizing potential confounding from major covariates. Employment status (*p* = 0.97), income level (*p* = 0.61), smoking prevalence (*p* = 0.98), and alcohol intake (*p* = 0.99) were all similarly distributed across groups, indicating baseline homogeneity of key lifestyle and socioeconomic variables (17–23). Mean body weight did not differ significantly among groups (*p* = 0.28). However, BMI (*p* = 0.03) and waist circumference (*p* = 0.04) showed statistically significant differences, both of which are important metabolic determinants in NAFLD. These variables were therefore adjusted in multivariable analyses to control for potential confounding effects. The ketogenic group exhibited slightly higher BMI (29.6 ± 3.8 kg/m^2^) and waist circumference (97.1 ± 8.6 cm), suggesting relatively greater central adiposity, a known predictor of hepatic steatosis and dyslipidemia (24–26). Blood pressure parameters showed no significant differences between groups (*p* > 0.05), consistent with prior NAFLD studies (27). Similarly, diabetes (*p* = 0.96) and hypertension prevalence (*p* = 0.99) were comparable across groups, indicating similar baseline cardiometabolic risk profiles (28, 29). Lipid profile analysis demonstrated significantly higher total cholesterol, triglycerides, and LDL-C in the ketogenic group (*p* < 0.001), as well as higher HDL-C compared with other groups (*p* < 0.001). These findings suggest diet-dependent alterations in lipoprotein metabolism, likely driven by differences in dietary fat intake and hepatic lipid handling (30–33). Liver enzyme levels (ALT and AST) were significantly higher in the ketogenic group (*p* < 0.01), while ALP remained unchanged (*p* = 0.21), indicating selective effects on hepatocellular injury pathways rather than cholestasis (34–36). Renal function markers (serum creatinine and eGFR) remained stable across groups (*p* > 0.05), suggesting no detectable short-term renal effect associated with dietary patterns (37–39). A multivariable regression analysis confirmed these associations after adjusting for confounders. The ketogenic diet was associated with an increase in total cholesterol (*β* = +62.9 mg/dL; 95% CI: 54.9 to 70.9; *p* < 0.001), while the low-fat diet showed a non-significant reduction (*β* = −5.1 mg/dL; 95% CI: −12.3 to 2.1; *p* = 0.16). Triglycerides were significantly increased in the ketogenic group (*β* = +36.5 mg/dL; 95% CI: 26.0 to 47.0; *p* < 0.001), with no significant change in the low-fat group (*p* = 0.49). LDL-C was significantly elevated in the ketogenic group (*β* = +34.2 mg/dL; 95% CI: 26.7 to 41.7; *p* < 0.001), while HDL-C also increased (*β* = +5.6 mg/dL; 95% CI: 3.8 to 7.4; *p* < 0.001). In contrast, the low-fat diet was associated with a modest reduction in HDL-C (*β* = −2.1 mg/dL; *p* = 0.01). ALT and AST levels were significantly increased in the ketogenic group (ALT: *β* = +8.7 U/L; AST: *β* = +5.9 U/L; both *p* < 0.01), whereas no significant changes were observed in the low-fat group. No statistically significant associations were observed between dietary patterns and renal outcomes (all *p* > 0.6), indicating no measurable effect of macronutrient distribution on short-term kidney function in this cohort (40).

### 4.1 Limitations

The retrospective cross-sectional design, with reliance on self-reported dietary data without objective adherence or physical activity measures, and the use of a sample from Baiyun District People’s Hospital and its affiliated campuses limit causal inference and generalizability. Baseline differences in BMI and waist circumference, key metabolic determinants in NAFLD, may introduce residual confounding despite statistical adjustment. Dietary assessment using FFQs and recalls is subject to recall bias, although repeated clinical records showed higher reliability. Furthermore, the observational nature of the study precludes causal conclusions, and reverse causality cannot be excluded. The absence of inflammatory biomarkers and subgroup analyses may also limit mechanistic interpretation and the overall depth of these findings.

## 5 Conclusion

Dietary patterns were significantly associated with variations in lipid metabolism and liver biochemical markers. The ketogenic diet was associated with higher levels of both HDL-C and atherogenic lipids, as well as elevated liver enzymes, while low-fat and balanced diets showed comparatively stable profiles. Renal function remained similar across groups. These findings suggest associations rather than causal relationships, highlighting the importance of individualized dietary guidance and careful metabolic monitoring.

## Data Availability

The original contributions presented in the study are included in the article/supplementary material, further inquiries can be directed to the corresponding author.
